# Life-Threatening Fungal Infection in Richter Transformation of Chronic Lymphocytic Leukemia/Small Lymphocytic Lymphoma: A Case Report and Brief Review of Literature

**DOI:** 10.7759/cureus.15924

**Published:** 2021-06-25

**Authors:** Binav Baral, Kriti Ahuja, Navika Chhabra, Muhammad J Tariq, Maryam Zia

**Affiliations:** 1 Internal Medicine, John H. Stroger, Jr. Hospital of Cook County, Chicago, USA; 2 Hematology and Oncology, University of Arizona, Tucson, USA; 3 Hematology and Medical Oncology, John H. Stroger, Jr. Hospital of Cook County, Chicago, USA

**Keywords:** chronic lymphocytic leukemia (cll), small lymphocytic lymphoma (sll), richter transformation (rt), diffuse large b cell lymphoma (dlbcl), invasive fungal infections

## Abstract

Chronic lymphocytic leukemia/small lymphocytic lymphoma is an indolent B cell lymphoproliferative malignancy typically affecting the elderly. Clinical outcomes of this condition have steadily improved as a result of advances in therapy. However, an increase in survival is accompanied by increased incidence of Richter transformation into an aggressive lymphoma. We present one such case and delve into its risk factors and associated complications. Exposure to increased lines of treatment appears to be a contributing factor in transformation, such that those with fewer than two lines of treatment are considered to have a lower risk of transformation. Fever, rapid lymph node involvement and drastic increases in lactate dehydrogenase, as seen in our patient, are concerning for transformation and treatment options include chemotherapy versus novel agent therapy. However, patients receiving therapy are at risk for adverse outcomes such as invasive infections, particularly in those receiving consolidation chemotherapy. Fungal infections such as Aspergillus and Candida are gaining prominence in the setting of neutropenia which adversely impact survival, but are underreported. Efforts to improve outcomes may include consideration of growth factor therapy in neutropenic patients and continuing to be vigilant for early signs of infection.

## Introduction

Chronic lymphocytic leukemia (CLL)/small lymphocytic lymphoma (SLL) is an indolent type of non-Hodgkin lymphoma, with progressive increase in survival secondary to advances in treatment [1-3]. With this increase in survival, the incidences of complications such as Richter transformation i.e., transformation into a high grade lymphoma have been observed more frequently, underscoring the importance of a deeper understanding on this phenomenon [4,5]. Possible complications associated with this condition include invasive fungal infections as a sequela of disease or chemotherapy. However, they are infrequent and have been underreported. Here, we present a case of Richter transformation complicated by life-threatening invasive fungal infection.

## Case presentation

**Figure 1:**
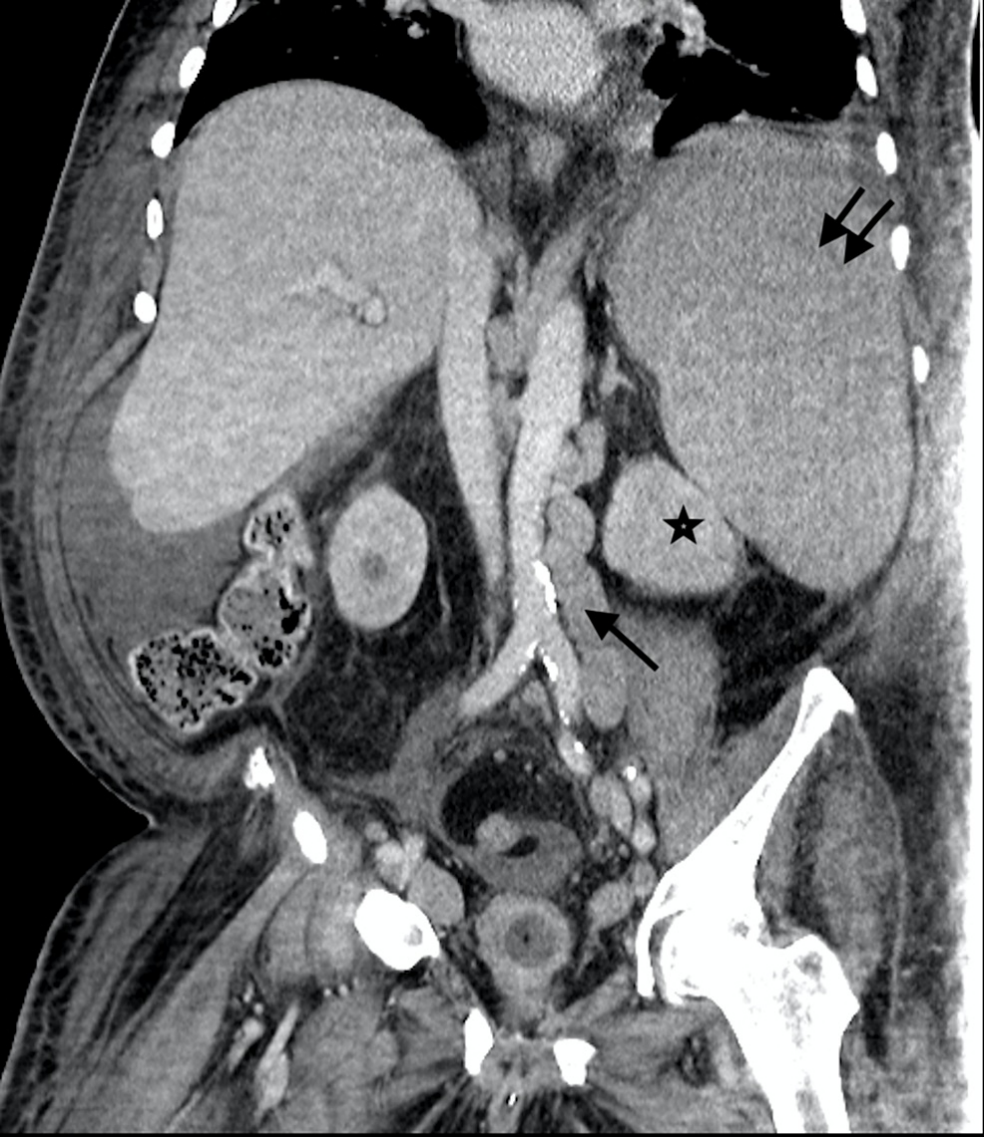
Coronal CT abdomen showing extensive para-aortic lymphadenopathy (arrow), with a large necrotic lymph node (star) and massive splenomegaly (double arrows).

**Figure 2:**
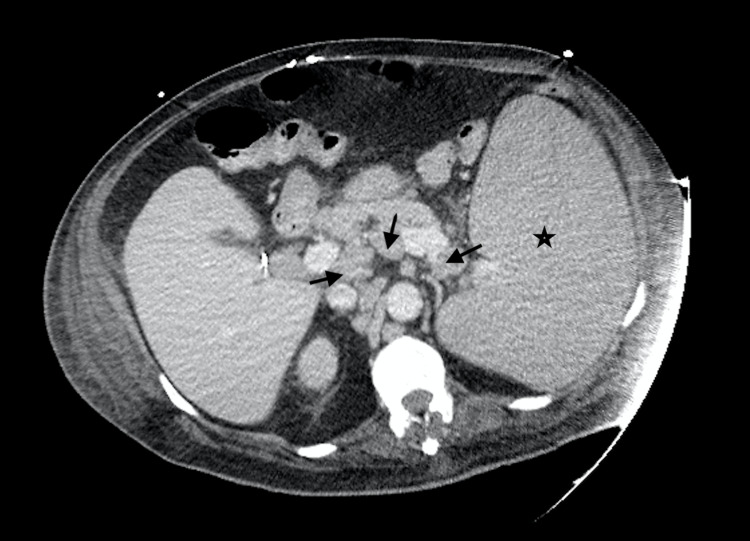
Axial CT abdomen redemonstrating generalized lymphadenopathy (arrows) with massive splenomegaly (star).

**Figure 3:**
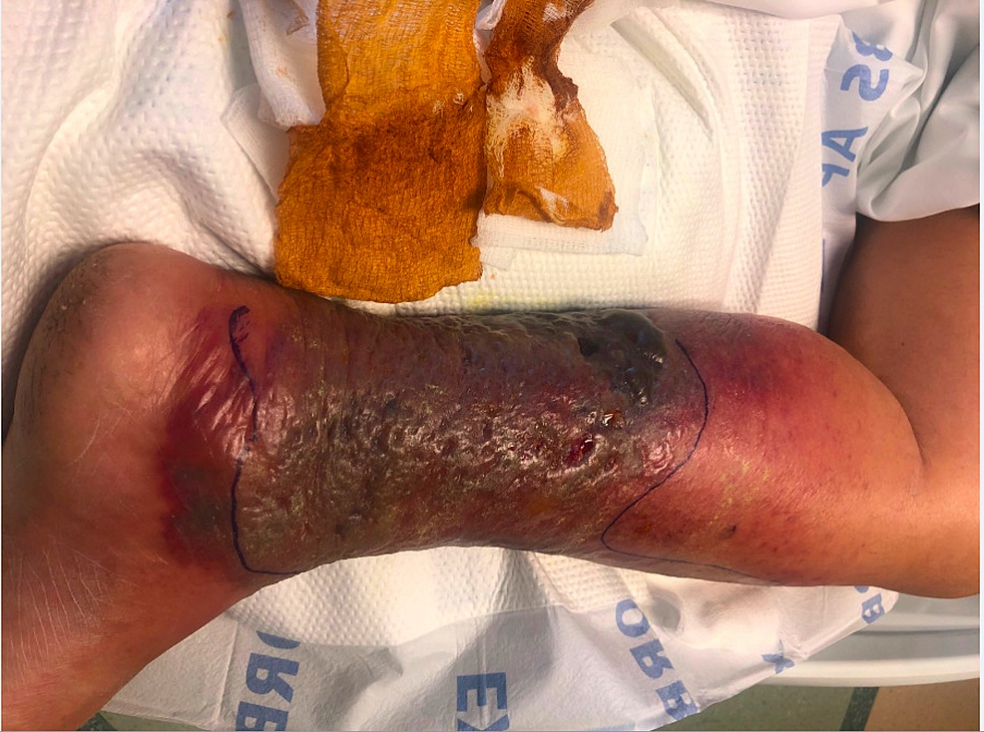
Fungal necrotizing hemorrhagic cellulitis of left lower extremity.

**Figure 4:**
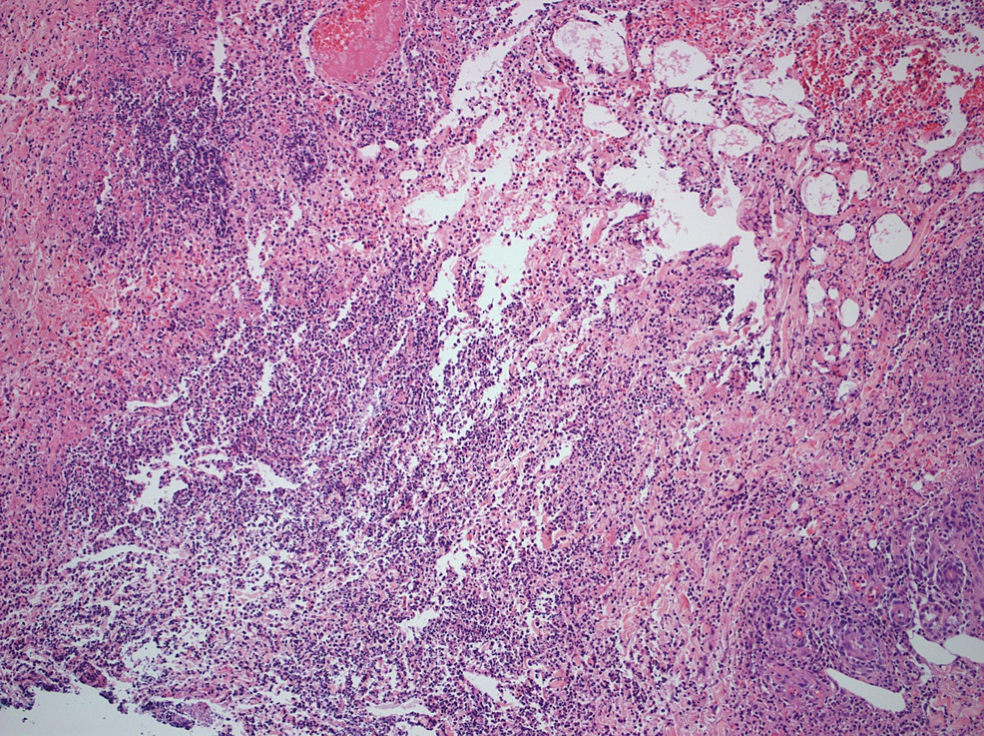
Histopathology specimen from dermal biopsy (100x magnification) showing skin with epidermal necrosis, superficial ulcer, prominent acute and chronic inflammation extending to subcutaneous tissue, abscess formation and thrombosis.

**Figure 5:**
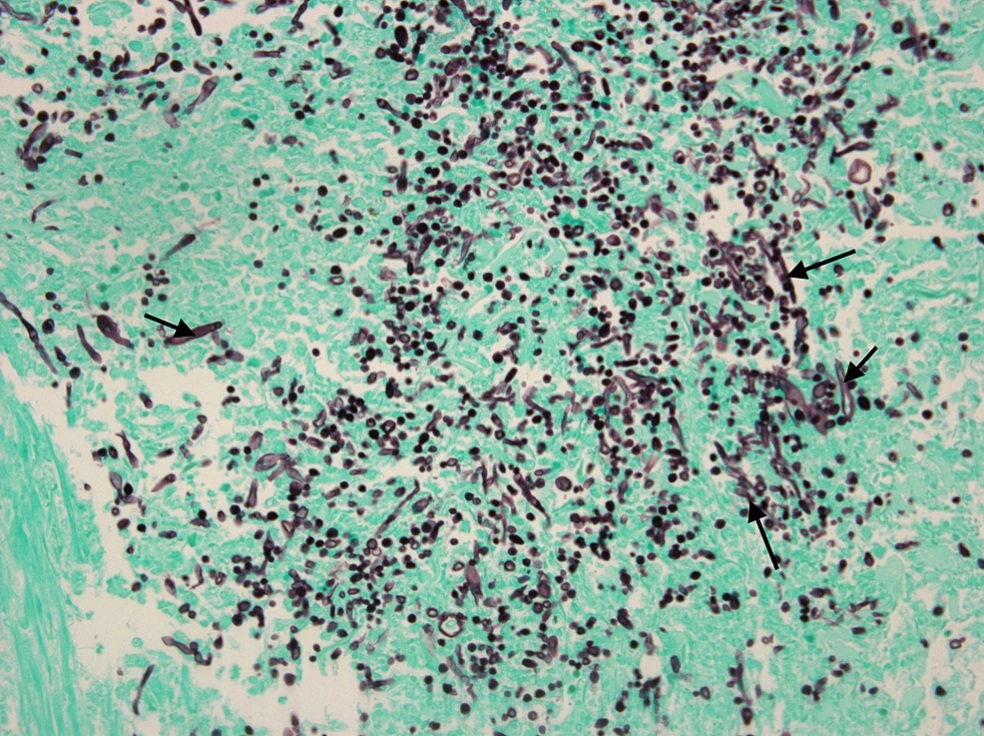
Microscopy of dermal biopsy (in 400x magnification) with Grocott-Gomori's methenamine silver (GMS) stain demonstrating numerous fungal elements containing large septated hyphae and budding yeasts (arrows).

We present a 68-year-old male with chronic lymphocytic leukemia (CLL)/small lymphocytic lymphoma (SLL) RAI I diagnosed at outside hospital by left axillary lymph node core needle biopsy, treated with six cycles of bendamustine and rituximab. He was lost to follow-up for four years, after which he presented with subjective fevers, night sweats, fatigue, pruritus, transient visual disturbances and myalgias. Examination revealed increase in the size of left cervical and supraclavicular lymph nodes with a stable left axillary lymph node. His labs were remarkable for a new anemia and worsening thrombocytopenia in three months with a hemoglobin decrease from 16.2g/dL to 8.5g/dL and platelets from 123,000/µL to 92,000/µL, respectively. Serum lactate dehydrogenase (LDH) level was 1100U/L with positive direct Coombs test, however reticulocyte count was 1.7%. Computerized tomography demonstrated extensive generalized lymphadenopathy with hepatosplenomegaly (Figures 1, 2). He underwent a repeat bone marrow biopsy which was consistent with CD5 negative low-grade B-cell lymphoma with deletion of 17p and TP53 mutation. The left supraclavicular node was also biopsied which showed double expressor (DEL; c-MYC+/BCL-2+) non-germinal center focal high-grade lymphoma with strong expression of CD5, arising from atypical small lymphocytic lymphoma (aSLL; CD23-, partial CD5+, 10% p53+), with enlarged proliferation centers, suggestive of Richter transformation with a Ki-67 index of 60-70%. He was then started on chemotherapy with R-CHOP (rituximab, cyclophosphamide, doxorubicin, vincristine, prednisone). However, after one cycle of chemotherapy, the patient developed neutropenic sepsis from hospital acquired pneumonia. This was further complicated by dermal biopsy-proven necrotizing hemorrhagic fungal cellulitis of left lower extremity (Figures 3-5) requiring above-knee amputation. He continued to deteriorate clinically despite treatment, devolving into septic shock and died one month after his first and only dose of chemotherapy.

## Discussion

Chronic lymphocytic leukemia (CLL)/small lymphocytic lymphoma (SLL) are B-cell lymphoproliferative malignancies of the elderly and are named as such based on the location of the malignant cells, in the blood and lymph nodes, respectively [1, 2]. CLL contributes to approximately a quarter of new leukemia cases, with the mean age at diagnosis being 70 years and typically following a stable course [3]. Occasionally, however, they can undergo transformation to more aggressive lymphomas (usually diffuse large B cell lymphoma (DLBCL), and rarely Hodgkin lymphoma), which portend a poor prognosis [4,5], known as Richter transformation. Common predisposing factors for this transformation include CD38 expression (≥30%), stereotyped B cell receptor, TP 53 disruption, NOTCH1 abnormalities, shortened telomeres (<5000bp), enlarged lymph nodes (>3cm), non-deletion 13q cytogenetics, IGHV4-39 gene usage [4,5]. Clinical manifestations include fever, rapid growth of lymph nodes, and labs may be significant for sudden and severe LDH elevation [2,5]. In CLL/SLL patients with DLBCL transformation, clonal relationship to CLL clones is an adverse prognostic factor [5], while good performance status, platelets ≥ 100,000, LDH ≤ 1.5 times ULN (upper limits of normal), tumor dimensions ≤ 5cm and less than two prior therapies confer a good prognosis, all of which are components of the Richter score [4-7].

Regarding therapeutic options for transformed CLL/SLL, ibrutinib is a targeted therapeutic option which may be used to treat active disease in patients of all ages with TP53 mutations and/or 17p deletions, by its mechanism of Bruton kinase inhibition, until disease progression occurs [2]. Interestingly, a recent study demonstrated that even temporarily interrupting ibrutinib for procedures (such as colonoscopies) and surgeries may cause an abrupt release of inhibition of B-cell receptor signaling, resulting in Richter transformation. Fortunately, resumption of ibrutinib is associated with a favorable response in these patients with no recurrence of DLBCL up to 32 months of follow-up [8].

Further, a review of existing literature reveals that while targeted oral therapy has significantly improved the management of CLL, patients continue to undergo disease progression, with as many as one-fifth of previously treated patients undergoing Richter transformation [9,10]. It is possible that as we see improved survival in CLL/SLL, we will encounter Richter transformation more frequently. A contributing factor may be the increased number of therapeutic options as patients who have received more lines of treatment have been found to be at increased risk for transformation [11]. In keeping with this, a recent study revealed that Richter transformation was associated with better outcomes in CLL patients who were treatment-naïve, in comparison to patients who had received prior therapy (median OS 6.16 years vs 1.49 years) [12].

Unlike CLL/SLL, where immediate therapy may not always be indicated, once a patient undergoes Richter transformation, treatment is essential. Possible therapeutic approaches include chemotherapy (possibly followed by stem cell transplant), and novel agent therapy. Some of the chemotherapy regimens studied are R-CHOP (rituximab, cyclophosphamide, doxorubicin, vincristine, prednisone), ofatumumab + CHOP (cyclophosphamide, doxorubicin, vincristine, prednisone), R-EPOCH (rituximab, etoposide, prednisone, vincristine, cyclophosphamide, doxorubicin), OFAR (oxaliplatin, fludarabine, cytarabine, rituximab), Hyper-CVAD (cyclophosphamide, vincristine, doxorubicin and dexamethasone), among others. Common toxicities encountered with these regimens are bone marrow suppression and infections [5]. Our patient received R-CHOP, which has a response rate of roughly 67%, with complete response in 7% patients, and associated infections in 28% cases, as opposed to Hyper-CVAD, which has a response rate of 41%, complete response in 38% with a higher rate of infections (affecting half the patients). Novel agent therapy is also under investigation, with some possible options targeting the Bruton tyrosine kinase pathway (ibrutinib, acalabrutinib), bcl-2 inhibition (venetoclax) among others [5].

As therapies for hematological malignancies advance, their adverse effects must be considered for early intervention and improved outcomes. Infection is an important complication of chemotherapy, and fungal infections are gaining prominence. Hence, it is increasingly relevant for the general physician to have a high clinical suspicion and provide timely and appropriate therapeutic coverage for fungal infections. CLL, as an indolent disease, has a low risk for invasive fungal infections (approximately 1.3%) of which yeast infections are more common than molds (1% vs 0.3%). Noted therapy-related factors include stem cell transplantation within the last 100 days, neutropenia (<500/mmc) and steroid therapy. Furthermore, a higher risk is observed with consolidation therapy, such that young high-risk DLBCL patients who were receiving consolidation therapy (R-MICMA (rituximab, mitoxantrone, carboplatinum, methylprednisolone and aracytin) and hematopoietic stem cell transplantation) are more likely to develop invasive fungal infections than young standard-risk DLBCL who received R-CHOP without consolidation therapy. Interestingly, even elderly DLBCL patients were not found to be at increased risk for invasive fungal infections if they were not recipients of intensive consolidation therapy [13].

A recent study demonstrated significantly increased risk of developing aspergillus and Candida infections in those who received therapy for CLL, including agents such as rituximab, chlorambucil, fludarabine and bendamustine [14]. Certain varieties of Candida are known to occur more frequently in hematological malignancies, such as Candida tropicalis (as was observed in our patient), Candida glabrata and Candida krusei [15]. Dendle et al. looked at DLBCL patients receiving R-CHOP and R-CHOP like therapy over a 10-year period from 2004 to 2014 and found that approximately two-thirds of the patients had at least one infection, most commonly involving the lower respiratory tract, followed by skin and soft tissue infections and bloodstream infections. Fungal infections accounted for one-fifth of the infections, with Candida being the causative organism in over 80% cases, half of which were oral thrush [16]. However, reporting of invasive fungal cellulitis in this subset of patients has been infrequent. Our patient received R-CHOP therapy, resulting in neutropenia, subsequently developed candidal cellulitis unresponsive to medical management and ultimately required an above-knee amputation for source control. While chemotherapy by itself suppresses immunity, predisposing patients to infections, the use of steroids as part of the chemotherapy regimen may further increase vulnerability. Neutropenia significantly increases risk for infection and has been found to lower survival. Dendle et al. also observed that those who had not received growth factor support within the past 21 days were more likely to have an infection, which may be explained by the improvement in white cell count leading to improved clearance of infections. This benefit of growth factor did not, however, translate into improved survival. Despite the risk for complications of therapy, however, over the course of their 10-year study of DLBCL patients, they found that while infections contributed to approximately 12% of the mortality, half the mortality was due to progressive lymphoma. This reinforces the rationale of treating Richter transformation despite the risk of complications with therapy, instead of adopting a watch-and-wait approach [16].

## Conclusions

CLL/SLL has a relatively stable course, with improved survival due to increased therapeutic options. As patients receive more lines of treatment, however, we must prepare to encounter complications such as Richter transformation more frequently. In the current scenario, the management for Richter transformation involves chemotherapy, with tendency for neutropenia and associated infections threatening patient survival. Fungal infections, although rare, can be life-threatening. Hence, to improve outcomes, physicians may consider growth factor support for neutropenia with filgrastim and remain vigilant for early signs of infection to provide adequate antimicrobial coverage. With increasing prevalence of fungal infections in high-risk patients, a strong index of suspicion and judicious use of antifungals is crucial.
